# DES-TOMATO: A Knowledge Exploration System Focused On Tomato Species

**DOI:** 10.1038/s41598-017-05448-0

**Published:** 2017-07-20

**Authors:** Adil Salhi, Sónia Negrão, Magbubah Essack, Mitchell J. L. Morton, Salim Bougouffa, Rozaimi Razali, Aleksandar Radovanovic, Benoit Marchand, Maxat Kulmanov, Robert Hoehndorf, Mark Tester, Vladimir B. Bajic

**Affiliations:** 1 King Abdullah University of Science and Technology (KAUST), Computational Bioscience Research Center (CBRC), Thuwal, 23955-6900 Saudi Arabia; 2 King Abdullah University of Science and Technology (KAUST), Division of Biological and Environmental Sciences and Engineering, Thuwal, 23955-6900 Saudi Arabia; 3 New York University, Abu Dhabi, UAE; 4 King Abdullah University of Science and Technology (KAUST), Computer, Electrical and Mathematical Sciences and Engineering Division (CEMSE), Thuwal, 23955-6900 Saudi Arabia

## Abstract

Tomato is the most economically important horticultural crop used as a model to study plant biology and particularly fruit development. Knowledge obtained from tomato research initiated improvements in tomato and, being transferrable to other such economically important crops, has led to a surge of tomato-related research and published literature. We developed DES-TOMATO knowledgebase (KB) for exploration of information related to tomato. Information exploration is enabled through terms from 26 dictionaries and combination of these terms. To illustrate the utility of DES-TOMATO, we provide several examples how one can efficiently use this KB to retrieve known or potentially novel information. DES-TOMATO is free for academic and nonprofit users and can be accessed at http://cbrc.kaust.edu.sa/des_tomato/, using any of the mainstream web browsers, including Firefox, Safari and Chrome.

## Introduction

The Solanaceae family is a major plant family comprising several economically important crop species such as potato (*Solanum tuberosum*), eggplant (*Solanum melongena*), tomato (*Solanum lycopersicum*), peppers (*Capsicum annuum*) and chili peppers (*Capsicum frutescens*). Globally, cultivated tomato is the most important horticultural crop, with an annual production of approximately 164 million tons, and with a value of about $US 60 bn (FAOSTAT, 2013). Because of its value as a food source, tomato has been a target for crop breeding programs focused on traits that contribute to lower production costs, higher quality fruit with extended shelf-life, and sustainable production with higher yield^1^. Tomato, like many other domesticated crops, has suffered a drastic erosion of genetic variation. Thus, wild tomato species have been widely used in breeding programs to increase genetic variation especially for stress tolerance^1, 2^. All of the 13 known wild tomato species^3, 4^ are diploid, can be crossed with cultivated tomato and are important for the evolutionary history of the *Solanum* section *Lycopersicon* clade^5–7^. Due to tomato’s unique features, such as its sympodial shoot, compound leaves, and fleshy fruit, this species has become an established model to study plant biology and particularly fruit development^8^.

The availability of the reference genome of *S*. *lycopersicum* ‘Heinz 1706’^9^, the identification of millions of single-nucleotide polymorphisms (SNPs)^10–12^, and the launch of the ‘150 tomato genome re-sequencing project’ (http://www.tomatogenome.net/)^5^ together with the SNP data from other 360 tomato accessions^13^, have paved the way for a myriad of genomic studies in tomato and its wild relatives. The large volume of data generated from these studies further prompt the development of tomato-related resources such as the TOMATOMICS database (http://bioinf.mind.meiji.ac.jp/tomatomics/index.php)^14^, the Micro-Tom mutant database - TOMATOMA (http://tomatoma.nbrp.jp/)^15^, and the Plant Omics Data Center (PODC; http://bioinf.mind.meiji.ac.jp/podc/)^16^, which includes core gene expression information for tomato and other species. The tomato-related research addresses different topics, such as stress tolerance^17, 18^, plant-pathogen interactions^19^, transcriptional control of biological processes^20, 21^ and fruit biology^22, 23^. This plethora of information is becoming overwhelming. Thus, proper insights into metadata are critical to allow a straightforward way to analyze and establish associations within tomato-related literature. The Sol Genomics Network (SGN) (http://solgenomics.net)^24^ presents a clade-oriented database for the Solanaceae family. However, the SGN is only able to retrieve information previously curated by the Solanaceae consortium, such as genes, quantitative trait loci, and computationally predicted gene family members. As such, the most appealing ingredient in the SGN, which is manual curation, is also its most limiting aspect. The SGN depends on past curation and therefore can only capture a small part of information.

Several text-mining derived knowledgebases (KBs) that explore topic-specific literature and focus on “term associations”/“terms co-occurrence”, have been developed for life science topics^25–39^. Text-mining in these KBs is restricted to titles and abstracts from PubMed records, which is beneficial for extracting a significant portion of useful information. However, the increasing availability of full-text articles in electronic form is expanding sources of information. For example, when comparing the distribution of information contained in full-text articles versus abstracts, Shah, *et al*.^40^ recommended the use of full-text articles instead of just abstracts for extraction of keywords. Also, Schuemie, *et al*.^41^, who reported that, although abstracts have the highest information density, results sections have the highest information coverage. In plant sciences, however, text-mining has not been fully exploited^35^. These include, for example, textual data on Arabidopsis in combination with an integrated network approach^42^, the Ondex data integration platform (http://www.ondex.org/index.shtml), designed to identify key protein-stress associations^43^, and VESPA mining, a platform to access data information contained in documents (in this case printed bulletins) to explore pest and crops interactions^44^. In addition, HRGRN resource (http://plantgrn.noble.org/hrgrn/) enables the exploration of regulatory networks in Arabidopsis (i.e. signaling transduction, metabolism and gene regulation) through a graph search-empowered integrative database^45^. Nevertheless, and while effective in identifying topic-specific associations, the previous use of text-mining in plant sciences, to our knowledge, tends to have a relatively narrow scope.

To enable users to make a more thorough exploration of the information related to tomato and its close relatives, we developed a topic-specific KB, DES-TOMATO, with an upgraded text-mining methodology similar to^46^. Our KB uses a dictionary-based approach in which enriched terms and phrases (referred to as terms from here on) belonging to different thematic categories (e.g. pathways, genes, taxonomy, etc.) are pre-compiled to form the basis for indexing text. Terms can be atomic, when the data source provides only one name variation for the entity in question, or they can have a number of synonymous words/phrases that are normalized to the same internal identifier within our knowledgebase. These internal identifiers allow for the universal identification of the term (e.g. through its EntrezGene gene ID, NCBI Taxonomy ID, etc.), and for complementing text-mined information with data from external sources if needed. This dictionary approach allows the user to focus on entities of their interest as defined from commonly used authoritative sources such as ChEBI^47^ and EntrezGene^48^. Our KB aims to discover associations between enriched terms, where these terms are searched for in titles and abstracts (from PubMed Wheeler, *et al*.^49^) as well as full-length articles allowed for text-mining (from PubMed Central Wheeler, *et al*.^49^). Moreover, due to the importance of tomato as a model for the study of plant-pathogen interactions, relevant dictionaries have been included so that users can explore the tomato-associated viral, bacterial, archaeal, and fungal species, as well as their genes and pathways involved in the biotic stress response. The KB also enables users to explore abiotic stress responses.

DES-TOMATO is a resource designed to assist in the exploration, analysis and discovery of tomato-related information inferred through the integration of several data sources. We demonstrate the effectiveness of DES-TOMATO in finding useful associations by presenting four case studies. These examples demonstrate how users can, with ease and speed, identify putative candidate genes, build a network of gene regulation for a specific trait, generate topic-specific hypotheses and explore enriched pathways. To our knowledge, this is the only KB derived through literature text-mining that has a comprehensive information exploration capabilities dedicated to the *Lycopersicon* section of the Solanaceae.

## Systems and Methods

DES-TOMATO is a topic-specific literature exploration system, designed to be visual, intuitive and interactive, and was generated using the Dragon Exploration System v2.0 (DES v2.0). DES was originally developed by VBB and AR and subsequently improved in various ways.

The knowledgebase is implemented and hosted on a CentOS-7 operating system. It uses Apache 2.4.6 as a web server. The literature repository is hosted on a MongoDB 2.6.11 database, and the KB index and related tables are hosted on a PostgreSQL 9.2.15 database. DES-TOMATO uses a Lucene text index for fast querying of the literature. Different components of the KB were developed using various programming languages/tools, namely: Java (openjdk 1.8.0_91), C/C++ (gcc 4.8.5), Perl v5.16.3, PHP 5.4.16, JavaScript, and JQuery 3.0.0.

DES-TOMATO is functional across major web-browsers on Linux, Windows, and Mac OS platforms. It was specifically tested for Firefox, Chrome and Safari. The only feature that we are aware of, which is functional only on Firefox, is the network export function. DES-TOMATO was not tested for hand-held devices, and is not currently intended for such use.

**Figure 1:**
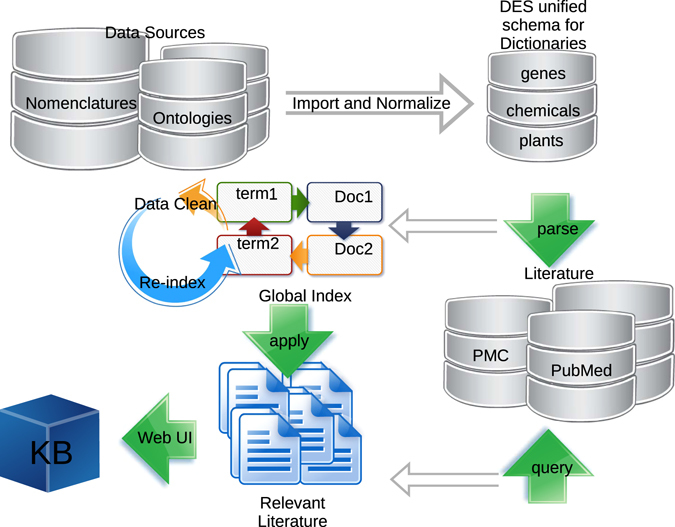
Workflow used within DES to create a KB such as DES-TOMATO.

The workflow used within DES to create a KB such as DES-TOMATO comprises the following steps (Fig. 1): 1/data imports and normalization into DES unified schema for dictionaries; 2/indexing of literature repositories using the said dictionaries, and using the resulting index for preliminary data cleaning; 3/preparation of literature corpus via querying of PubMed and PubMed Central articles; 4/extracting term-document mapping information from the global index (created in step 2) that are specific to the corpus in context (defined in step 3); 5/creation of the KB by applying various analysis tasks, including statistical enrichment of terms, extraction and enrichment of pairs, and integrating these data with relevant external resources.

### Preparing the literature corpus

To create DES-TOMATO, we first queried our local literature repository, a MongoDB repository hosting PubMed and PubMed Central articles, backed up by a Lucene text index for fast query servicing. The following DES-TOMATO query was used to incorporate all tomato species: [tomato* OR lycopersicum OR lycopersicon OR ((Solanum OR S.) AND (esculentum OR pimpinellifolium OR pennellii OR sitiens OR habrochaites OR neorickii OR cheesmaniae OR galapagense OR peruvianum OR arcanum OR chilense OR huaylasense OR juglandifolium)]. This retrieved 22,647 articles. The query was made on data updated on August 30, 2016.

### Terms and dictionaries

Terms are compiled into thematic dictionaries. Terms can be atomic, when the data source provides only one name variation for the entity in question, or they can have a number of synonymous words/phrases that are normalized to the same internal identifier within our knowledgebase. These internal identifiers allow for the universal identification of the term (e.g. through its EntrezGene gene ID, NCBI Taxonomy ID, etc.), and for complementing text-mined information with data from external sources if needed.

Regarding the dictionaries of genes, we combine EntrezGene nomenclature (for genes) with UniProt nomenclature (for proteins) for a number of reasons. In literature, gene names or symbols are frequently used interchangeably with the names or symbol of their products. Thus, we also use UniProt nomenclature. These nomenclatures provide naming conventions that are the most used by the biomedical community in literature. When reporting results related to a particular gene/protein, it is customary to use the official name/symbol of the gene/protein or one of its aliases and EntrezGene and UniProt exhaustively provide these. EntrezGene also provides loci names for genes as unique identifiers within a species, which are also heavily used in text. There is an initiative by the Tomato Genome Consortium^50^ to introduce a standardized annotation for gene loci following *Arabidopsis* type identifiers, that is, these loci names have a general format of the type such as Solyc00g005440.1. Although we intended to use these identifiers, our search for ‘Solyc’ type identifiers in the whole of PubMed produced no hits, which may be partly due to their relatively recent adoption.

Dictionary selection and curation is one of the most important tasks in our KB building process. To ensure relevance and comprehensiveness, we imported 19 relevant dictionaries from the pre-existing DES v2.0 vocabularies. Furthermore, we compiled seven additional theme-specific dictionaries, namely: “Stress-related Vocabulary”, “Plant-related Vocabulary”, “Green Plants Genes (EntrezGene)”, “Solanaceae Genes (EntrezGene)”, “Green Plants (NCBI Taxonomy)”, “Solanaceae (NCBI Taxonomy)” and “Tomato Species (NCBI Taxonomy)” (see Table 1 for more details).

**Table 1:** List of dictionaries used in DES-TOMATO.

Dictionary	Enriched Unique Terms in the KB	Source
**Chemicals/Compounds**		
Chemical Entities of Biological Interest (ChEBI)	4561	pre-existing in DES
Metabolites (MetaboLights)	1556	pre-existing in DES
Enzymes (IntEnz)	1182	pre-existing in DES
Toxins (T3DB)	886	pre-existing in DES
Antibiotics	244	pre-existing in DES
Industrially Important Enzymes (EC)	215	pre-existing in DES
**Functional Annotation**		
Pathways (KEGG, Reactome, UniPathway, PANTHER)	576	pre-existing in DES
Biological Process (GO)	1288	pre-existing in DES
Molecular Function (GO)	474	pre-existing in DES
Cellular Component (GO)	466	pre-existing in DES
**Genes/Proteins/Transcripts**		
Green Plants Genes (EntrezGene)	16579	newly compiled
Solanaceae Genes (EntrezGene)	2994	newly compiled
Bacteria Genes (EntrezGene)	2879	pre-existing in DES
Fungi Genes (EntrezGene)	2758	pre-existing in DES
Viruses Genes (EntrezGene)	971	pre-existing in DES
Archaea Genes (EntrezGene)	536	pre-existing in DES
**Taxonomy**		
Green Plants (NCBI Taxonomy)	5733	newly compiled
Fungi (NCBI Taxonomy)	2426	pre-existing in DES
Bacteria (NCBI Taxonomy)	1498	pre-existing in DES
Viruses (NCBI Taxonomy)	1109	pre-existing in DES
Solanaceae (NCBI Taxonomy)	297	newly compiled
Source Microbes for Antibiotics	113	pre-existing in DES
Archaea (NCBI Taxonomy)	40	pre-existing in DES
Tomato Species (NCBI Taxonomy)	15	newly compiled
**General**		
Plant-related Vocabulary	2688	newly compiled
Stress-related Vocabulary	759	newly compiled

References for the data sources indicated in Table 1 are as follows: ChEBI (Hastings *et al*.^47^), MetaboLights^113^, IntEnz^114^, T3DB^115^, Industrially Important Enzymes EC^116, 117^, GO^118^, KEGG^119^, Reactome^120^, PANTHER^121^, UniPathways^122^, EntrezGene^48^, NCBI Taxonomy^123^, KOBAS^52^.

The following is a description of the process of importing/generating data for compiling dictionaries:

#### The general case

Irrespective of how the dictionary is generated, the importing and integration of a new dictionary into DES typically includes the following steps:

- Transforming the vocabulary data into a format that adheres to our local Term schema. This schema includes:
  - a unique identifier for the term,
  - a concept identifier shared by synonymous terms but unique across concepts,
  - the English version of the term itself (so removing non-English nomenclatures if they exist), as well as
  - metadata about the term, such as description, source (e.g. PO), the ID used by the source, (PO:0025002 for *‘basal root’*), etc.
- This is then used to update the dictionary set with the new data. New entries are checked for term redundancies within the same dictionary, in which case they are unified into one term with multiple source IDs.
- An initial indexing is performed to see how the newly imported dictionaries match the literature, (e.g. which terms did actually have mentions, and how frequently across the whole PubMed and the whole (allowed for text-mining) PubMed Central documents). This information also provides the basis for dictionary cleaning, as it is often the case that promiscuous terms from thematic dictionaries appear with high false positive rates due to the high frequencies of their use usually as common English words. Such terms we generally have excluded. An example is term “content”: one of the synonyms for PATO:0000025.
- Once the dictionary data is cleaned, another re-indexing occurs so that the index and the subsequent analyses are built around reasonably clean dictionary data.

We eliminated ambiguous terms from the dictionaries where possible. The problem of ambiguous words that might blur the outcome of a search, is a well-known challenge in the field of text-mining and natural language processing, because it is inherent to language^51^. Even in manual analysis of text, human interpretation is the key to disambiguate the meaning. In the case of DES-TOMATO, this disambiguation is left to the user and his/her knowledge and skills. Furthermore, this problem is more relevant to some dictionaries (types of biological entities) than others, e.g. gene names/abbreviations coinciding with disease names/abbreviations, or some ontologies containing some semantically broad terms. However, to reduce the proportion of these cases in DES-TOMATO, we carried out stringent term pre-processing steps: 1/initial data cleaning of the most frequent promiscuous terms, 2/eliminating terms shorter than three characters that have no synonyms in the same document, and 3/statistical enrichment, which filters out an additional good proportion of common and highly promiscuous terms.

#### The “Plant-related Vocabulary”

The “Plant-related Vocabulary” incorporates terms from a number of ontologies (see Table 2), which in some cases (e.g. FLOPO) are in turn, partially or completely, composed of information from other ontologies.

**Table 2:** Plant-related ontologies used to compile the “Plant-related Vocabulary”.

Terms or Phrases	Definition
Enriched Terms	Biological terms or keywords (e.g. lycopene, peroxidase activity, *Solanum pimpinellifolium*, etc.) used to mine the literature and organized into thematic dictionaries
Enriched Term Pairs	Connection/association (possibly biological) between two terms that is inferred based on the co-occurrence of these terms (e.g. signaling and salicylic acid; lycopene and carotenoids; *Solanum lycopersicum* and begomovirus, etc.)
Hypothesis	New connection of terms; a starting point for possible further investigation (e.g. AGO5 and ‘DNA methylation’; SNI1 and ‘jasmonic acid’)
KOBAS Pathways	Enriched pathways that were identified by the set of genes and/or proteins extracted from tomato-based literature
Dictionary	A set of terms, which are categorized into themes (e.g. Pathways, Metabolites, or Genes)
**Interactive tools**	**Definition**
Network Viewer	A tool for the visualization of term associations as a graph of interlinked nodes
Term Co-occurrences	A list of all the enriched terms from all dictionaries that is potentially associated with the term in question.
Term Link Sources	A graph/pie chart that visualize the distribution of data sources (dictionaries) from which associations to the term in question are drawn

Note that sometimes ontologies reuse and integrate entities from other ontologies/sources when appropriate, such as is the case for FLOPO and PTO ontologies.

#### The “Stress-related Vocabulary”

This vocabulary was built from scratch to account for certain terminology that we believe is important for this KB, but was lacking in the plant ontologies that we considered. For compiling the “Stress-related Vocabulary”, we created 19 categories of keywords: ‘Salt’, ‘Heat’, ‘Cold’, ‘Flood’, ‘Drought’, ‘Light’, ‘pH’, ‘Osmotic’, ‘Oxidative’, ‘Anaerobia’, ‘Anoxia’, ‘Hypoxia’, ‘Hyperoxia’, ‘Nitrosative’, ‘Physiology’, ‘Nutrients’, ‘Pathology’, ‘Growth’, and ‘Biotic’. In each category, we manually searched the literature and added keywords that are related to the category, with the condition that the keyword must not exist in any plant ontology. For example, under the ‘Osmotic’ and ‘Flood’ categories, we included terms ‘Osmoprotectant’ and ‘Submergence’, respectively. These two keywords are related to stress and they are not found in any of the other DES-TOMATO dictionaries. In total, 92 keywords from the literature for the 19 categories were identified. Concurrently, we created 23 keywords that act as prefixes, such as ‘tolerance to’ (e.g. tolerance to salt stress) and 7 keywords that act as suffixes, such as ‘tolerance’ (e.g. salt stress tolerance). We then computationally compiled these affixes to the 92 keywords that resulted in 2,760 new terms that we used in the text-mining process. Some of these combinations were not detected in text, either because they were not used or because they do not representing viable term combinations.

### Post-processing and indexing

Terms in the aforementioned dictionaries were then mined in the retrieved articles, highlighted and color-coded according to dictionary. This process is enabled by the back-end index that matches terms to their occurrences, up to the character level, within the mined articles. In total, 9,499,592 terms from 26 dictionaries were used to index the literature corpus in DES-TOMATO. A term is defined as enriched when it is overrepresented in DES-TOMATO documents as compared to all PubMed and all PubMed Central articles (for which text-mining is allowed) from our local repository. We used a false discovery rate (FDR) < 0.05, which was calculated based on the Benjamini–Hochberg procedure to correct for multiplicity testing. Terms in all dictionaries are normalized, i.e. names, symbols and synonyms referring to the same concept are represented by a single entity when analyzed. This process allowed us to identify 52,886 unique terms that are statistically enriched (FDR <= 0.05) in tomato-related documents and present in DES-TOMATO. We further identified 1,388,952 enriched unique term pairs (FDR <= 0.05) formed from the 52,886 statistically enriched terms.

Additionally, by matching genes and proteins enriched in DES-TOMATO to other resources beyond the KB literature corpus, in this case KOBAS^52^, we found hits to: 1/930 Bacterial pathways, of which 677 are statistically enriched (FDR <= 0.05), 2/427 Archaeal pathways, of which 90 are statistically enriched (FDR <= 0.05), 3/523 Fungi pathways, of which 86 are statistically enriched (FDR <= 0.05), and 4/1,747 Plant pathways, of which 488 are statistically enriched (FDR <= 0.05).

## Results

### Indirect assessment of the quality of extracted information

It is difficult to provide a global assessment of the quality of extracted information by DES-TOMATO KB. In an attempt to provide an independent assessment of the quality of associations identified by KB, we evaluated the quality of the gene pairs extracted by the KB by comparing them to their functional similarity, where functions of the genes are obtained from an independent data source. Specifically, we computed the semantic similarity of gene pairs based on their GO annotations using the Semantic Measures Library (SML)^53^. We hypothesize that a strong correlation between our extracted associations between gene pairs and their functional similarity is reflective of the quality of the data in DES-TOMATO and its analysis approach. Essentially, we propose that a correct association between two genes in DES-TOMATO will generally be reflected by the two genes’ sharing similar GO annotations, although some gene pairs may also be associated in a manner not reflected by GO term similarities. In other words, we performed an assessment of the quality of extracted tomato gene-gene associations under strict conditions.

EntrezGene IDs for normalized genes were mapped to identifiers in the agriGO annotation^54^. Starting with a total of 16,056 Solanaceae gene pairs, we removed all gene pairs between genes that are in another Solanaceae species, and retained 13,139 pairs in which at least one of the genes is present in tomato. Selecting pairs in which both genes are present in tomato produced a set of 3,975 pairs of which 2,227 had an agriGO annotation for both genes in each pair. We use only these 2,227 pairs in the assessment by semantic similarity. Here we used default parameters (lin_resnik_bma) with the aspect parameter set to GLOBAL. Of the 2,227 tomato gene pairs, 575 (26%) had maximum possible semantic similarity (value of 1.0), which means that genes in these pairs have identical GO annotations. Table 3 lists some examples from this set. In Table 4, we show the percentage of identified pairs of genes at different semantic similarity thresholds.

**Table 3:** Examples of gene-gene associations identified in KB with semantic similarity equal to 1.0.

Gene Symbol/Description	Gene Symbol/Description	Common Annotations
SERK3A/ID: 100736467 somatic embryogenesis receptor kinase 3 A [*Solanum lycopersicum* (tomato)]	LOC101259548/ID: 101259548 leucine-rich repeat receptor-like serine/threonine/tyrosine-protein kinase SOBIR1 [*Solanum lycopersicum* (tomato)]	“protein kinase activity”;“molecular_function”;“GO:0004672” “protein binding”;“molecular_function”;“GO:0005515” “ATP binding”;“molecular_function”;“GO:0005524” “protein phosphorylation”;“biological_process”;“GO:0006468”
PHYF ID: 101259349 phytochrome F [*Solanum lycopersicum* (tomato)]	PHYB1 ID: 101262847 phytochrome B1 [*Solanum lycopersicum* (tomato)]	genes are involved in photoreceptor activity (GO:0009881)
APX2 ID: 778224 cytosolic ascorbate peroxidase 2 [*Solanum lycopersicum* (tomato)]	LOC101264261 ID: 101264261 L-ascorbate peroxidase 3, peroxisomal [*Solanum lycopersicum* (tomato)]	“peroxidase activity”;“molecular_function”;“GO:0004601” “peroxidase activity”;“molecular_function”;“GO:0004601” “response to oxidative stress”;“biological_process”;“GO:0006979” “heme binding”;“molecular_function”;“GO:0020037” “oxidation-reduction process”;“biological_process”;“GO:0055114”

**Table 4:** The change of the number of gene pairs according to the change of required semantic similarity level.

Semantic Similarity	Number of Gene Pairs	Percentage (out of 2,227)
>=0.4	1098	49%
>=0.45	991	45%
>=0.5	943	42%
>=0.55	913	41%
>=0.6	875	39%
>=0.65	832	37%
>=0.7	794	36%
>=0.75	760	34%
>=0.8	697	31%
>=0.85	674	30%
>=0.9	613	28%
>=0.95	579	26%
=1	575	26%

Furthermore, results shown in Supplementary Material (distribution of high similarity pairs across FDR rank) demonstrate that the higher the FDR rank of a gene pair, the more likely it would have a high similarity rank. This shows the usefulness of the enrichment measure we use in DES-TOMATO. Therefore, our system not only extracts gene pairs through co-occurrence, it also has a robust means for ranking, or prioritizing, these associations.

It is important to note that for a number of pairs suggested by DES-TOMATO it was not possible to calculate the similarity score due to either one or both of the tomato genes in the pair lacking GO annotation in agriGO (as mentioned above). These gene pairs, which were false positives in our stringent assessment, should not be considered as unrelated. In fact, we manually evaluated a number of these ‘inconclusive’ pairs and found that some do have an association that was not reflected in the semantic similarity (see examples in Table 5). Unfortunately, manual curation of the entire dataset is beyond our means.

**Table 5:** Some examples of gene-gene associations that have functional association but do not have semantic similarity.

Gene Symbol/Description	Gene Symbol/Description	Common Annotations	Reference
**IAA3**/ID: 543540 IAA3 protein [*Solanum lycopersicum* (tomato)]	**EXP2**/ID: 543582 expansin [*Solanum lycopersicum* (tomato)]	Volatile Organic Compounds (albuterol and 1,3- propanediole) were shown to promote lateral root formation that correlates with an increase in levels of EXP2 and IAA3 in the roots of tomato plants	124
**MAF1**/ID: 543586 MFP1 attachment factor 1 [*Solanum lycopersicum* (tomato)]	**FPP**/ID: 543699 filament-like plant protein [*Solanum lycopersicum* (tomato)]	Filament-like plant proteins (FPP) belongs to a family of long coiled-coil proteins that interacts with the nuclear envelope-associated protein, MAF1	125
**LOC543607**/ID: 543607 pirin [*Solanum lycopersicum* (tomato)]	**DAD1**/ID: 543753 dad-1 protein [*Solanum lycopersicum* (tomato)]	Both DAD1 and pirin are mediators of programmed cell death in plants. However, DAD1 was shown to interact with BCL2 family members, while pirin plays more of a downstream role as it forms a NF-kB, BCL3, Pirin complex that is capable of modulating NF-kB-driven gene expression through interaction with an NF-kB DNA-binding site.	126

Using one of the most challenging text-mining entities (genes/proteins), we have demonstrated that the quality of the associations in our KB is reasonably reliable and by extension we extrapolate that entities and associations in the other dictionaries in the KB are also reasonably reliable.

### Navigating the KB

The users of DES-TOMATO can explore and find relevant information in the literature, based on enriched terms. The content of this KB can be explored via links (described in detail by Salhi *et al*.^34^ under names in brackets), which include “Enriched Terms” [Concepts], “Enriched Term Pairs” [Associated Concepts], “Explore Hypotheses” [Hypothesis Explorer], and “KOBAS Pathways” [KOBAS pathways]. By navigating these links, users can view enriched terms via several types of ranking options and/or by restricting the FDR to zoom in on an enriched subset of interest. Moreover, users can access a menu with a right-click, which enables all terms to generate a “Network” view, “Term Co-occurrences” and “Term Link Sources” (refer to Table 6). It is important to note that users should always refer to organisms by their Latin name, namely for pathogens (except virus) and plant species. Case study examples are given below. We provided a detailed Manual that explains various functionalities of the DES-TOMATO and its use. Each page of the KB contains a link to “Help” for the fast instructions about how to use the page. In addition, we provided a quick start video on the “Home” page, which demonstrates basic functionalities of the KB.

**Table 6:** Glossary.

Ontology	Description
PO	**Plant Ontology** ^127, 128^: A structured vocabulary which incorporates: plant anatomy, morphology and growth and development. PO was developed as part of the Planteome project (License: http://planteome.org/License)
FLOPO	**Flora Phenotype Ontology** ^129^: an ontology of phenotypes reported in Floras. This ontology incorporates a number of entities from other ontologies, in addition to indigenous FLOPO entities.
PTO/TO	**Plant Trait Ontology** ^128^: A controlled vocabulary to describe phenotypic traits in plants. This ontology also incorporates classes from various other ontologies.
PECO/EO	**Plant Environmental Conditions Ontology** ^128^. This ontology describes the treatments, growing conditions, and/or study types used in plant biology experiments.
SPTO	**Solanaceae Phenotype Ontology** ^130^: Solanaceae crop phenotypes and traits, developed in collaboration with the research community, especially for breeder traits of agronomic importance.

### Case studies that substantiate the effectiveness of DES-TOMATO as a research supporting system

**Example 1**. “Enriched Terms” used for the *exploration of genetic interactions underlying bacterial speck disease*.

Here we explore the efficacy of DES-TOMATO in the exploration of plant-pathogen molecular interactions towards identifying the genetic components of resistance to bacterial speck (caused by *Pseudomonas syringae*) in the Solanaceae family. The genetic-basis for resistance to this disease was linked to the *Pto* gene^55, 56^.

**Figure 2:**
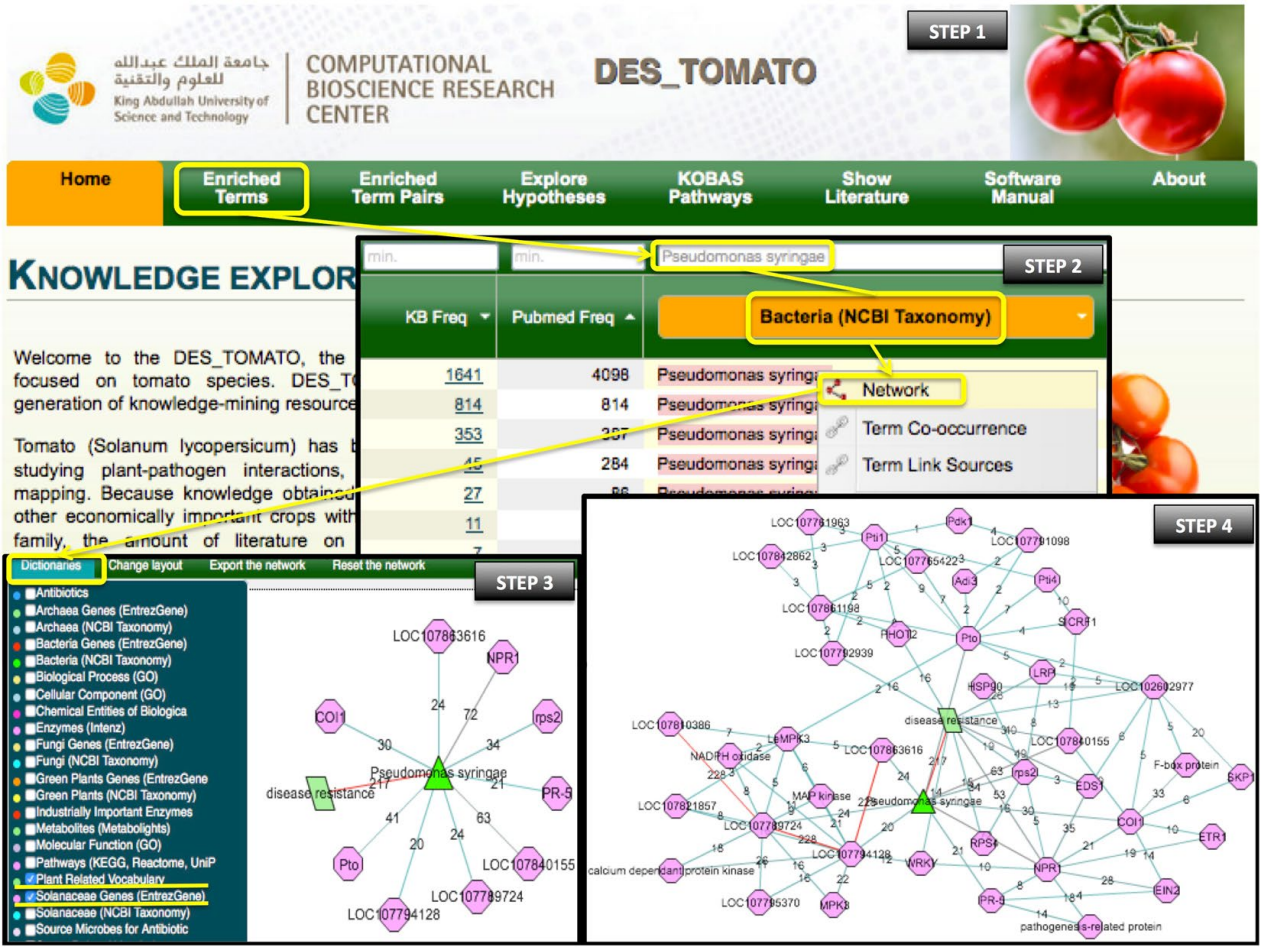
Step-by-step illustration of how DES-TOMATO can be used to identify components of genetic resistance for *P*. *syringae* (marked in yellow). The pink octagons represent the “Solanaceae Genes” dictionary; the dark green triangles represent the “Bacteria (NCBI Taxonomy)” dictionary; and the pale green trapezoids represent “Plant-related Vocabulary” dictionary. The edge color is distributed across a color spectrum from hot/red (high frequency co-occurrence/strong association) to cold/blue (small number of co-occurrences, weaker association). The numbers on the edges provide the number of publications that link the associated nodes.

We started exploring DES-TOMATO by clicking “Enriched Terms” (Fig. 2, Step 1), we, then, searched the list for ‘*Pseudomonas syringae*’, and generated a network with the right-click menu (Fig. 2, Step 2). On the network page, we selected “Solanaceae genes” and “Plant-related Vocabulary” from the dictionaries top-menu, then populated the network starting from the ‘*Pseudomonas syringae*’ node using the ‘Expand from the term’ right-click menu. Afterwards, we removed redundant terms, generic terms, and all “Plant-related Vocabulary” terms except ‘Disease resistance’ using the ‘Remove highlighted’ right-click menu (Fig. 2, Step 3). Using the “Solanaceae genes” dictionary only, a second round of network expansion was performed on all nodes obtained in Step 3, followed by a third round of expansion from the resulting ‘Pto’ node. The resulting network was simplified by removing nodes with a single link (Fig. 2, Step 4).

The final network is clearly divided into two sub-networks; one is centered on ‘Pto’ while the other is centered on ‘NPR1’ (Fig. 2), which is consistent with previous knowledge. Upon infection, Pto detects the cognate AvrPto bacterial effector proteins, triggering a signal transduction cascade^55, 57^. Additionally, it is known that Pdk1 regulates Adi3 activity together with Pto^58–60^, and the loss of Adi3-mediated cell death suppression is believed to contribute, through MAPKKKα signaling, to the resistance response upon *P*. *syringae* infection^61, 62^. Similar relevant connections can be made by expanding from the other *Pto*-associated genes (not shown).

On the other hand, NPR1 is a master immune regulator that indirectly drives transcription of PR genes in response to the immune signal salicylic acid (SA), eliciting a defense response^63^. Additionally, Coronatine-insensitive 1 (COI1), inhibits jasmonate (JA) signaling-dependent process that is known to impair SA-mediated pathogen defense responses^64^. This pathway is hijacked by various *P*. *syringae* strains expressing the phytotoxin coronatine (COR), which mimics a bioactive JA conjugate to suppress immune responses through interactions with COI1^65, 66^. Other noteworthy PRR genes associated with *P*. *syringae* include:

i) R gene Resistance to *P*. *syringae* 2 (*rps2*), which encodes an NB-LRR protein involved in the recognition of the *P*. *syringae* effector AvrRpt2^67, 68^;
ii) R gene Resistance to *P*. *syringae* (*rps4*), which cooperates with *Ralstonia solanacearum* 1 (RRS1), to recognize the *P*. *syringae* effector AvrRps4^69^; and
iii) Two PR genes, *PR5* and *PR1* (LOC107840155).

Through this example we demonstrate the ability of DES-TOMATO to effectively identify key factors underpinning systems of interest. In this case, DES-TOMATO enables the construction of complex networks representing the genetic interactions underlying plant-pathogen responses with relative ease and speed, with little prior knowledge. This approach identified many well-characterized components as well as less evident connections, such as the one between COI1 and SGT1 (only hypothesized in Meldau, *et al*.^70^, yet not experimentally shown), which can used as suggestions for future investigations.

**Example 2**. *“Enriched Term Pairs” used to explore “Na+/H+ antiporter” associated gene for the discovery of a putative candidate gene involved in salinity tolerance*.

**Figure 3:**
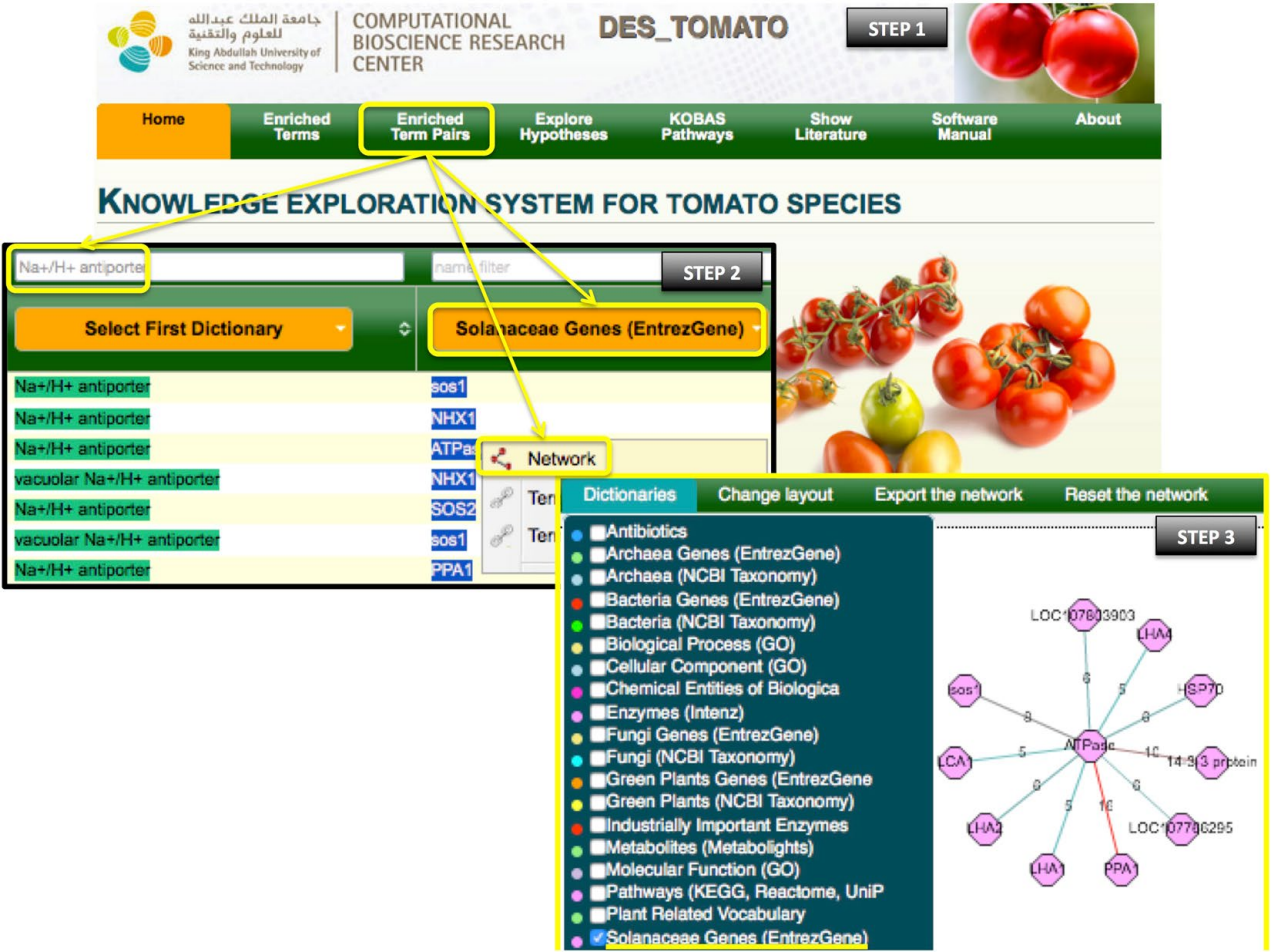
Step-by-step illustration of how DES-TOMATO can be used to find relevant candidate genes involved in salinity tolerance by focusing on Na^+^ homeostasis and plasma membrane H^+^-ATPases (in yellow). In the network, the pink octagons represent the “Solanaceae Genes” dictionary. The edge color is distributed across a color spectrum from hot/red (high frequency co-occurrence/strong association) to cold/blue (small number of co-occurrences, weaker association). The numbers on the edges provide the number of publications that link the associated nodes.

The accumulation of toxic levels of sodium in the cytosol is the main cause of salinity stress in plants, and cells cope through an efficient cytosolic Na^+^ homeostasis mechanism (e.g. Na+/H+ antiporters)^71^. To explore potential genes involved in this process, we start by clicking “Enriched Term Pairs” (Fig. 3, Step 1). This opens a page with two columns listing associated terms from all dictionaries. In the first dictionary (term A), we filtered the name for ‘Na+/H+ antiporter’ while in the second dictionary (term B), we selected the “Solanaceae genes” dictionary from the drop-down menu (Fig. 3, Step 2). The first two enriched term pairs are *SOS1* and *NHX1* genes, which are widely known in the literature to be involved in salinity response, meanwhile the third hit was ‘ATPase’. ATPases are proton pumps that are essential for establishing the proton gradient that powers the transport of Na^+^ by Na^+^/H^+^ antiporters across the plasma membrane and the tonoplast^71, 72^. Salinity stress induces the expression of H^+^-ATPases in both the tonoplast and the plasma membrane^73, 74^; thus, we chose to expand our search through ‘ATPase’. We right clicked on ‘ATPase’, and selected “Network” (Fig. 3, Step 2). In the new window, we selected ‘ATPase’ and expanded the association using the “Solanaceae Genes” dictionary (Fig. 3, Step 3). To focus the network, we removed redundant terms using the right click menu. Next, we searched PubMed for the other genes captured by the network and found the following:

i) LOC107803903, which encodes the ‘zinc transporter 5-like’ in *Nicotiana tabacum*.
ii) *HSP90*, which encodes the ‘Heat Shock Protein 90’ that has been reported to be involved in heat stress in tomato^75^;
iii) *HSP70*, which encodes the ‘Heat Shock Protein 70’ from *S*. *lycopersicum*. *HSP70* was proposed to act together with *HSP90*, at least, under heat stress^75^;
iv) LOC107766295, which encodes for the ‘Heat Shock cognate 70 kDa protein 2-like’ from *N*. *tabacum*;
v) *PPA1*, which encodes the soluble inorganic pyrophosphatase-like from *S*. *tuberosum*;
vi) 14-3-3 protein family, which is known to bind to several signaling proteins, namely activating the auto-inhibited plasma membrane H^+^-ATPases^76^;
vii) *SOS1*, which is a gene known to be involved in salinity response, and abundantly described in the tomato literature^77^;
viii) *LHA2*, which encodes for a plasma membrane H^+^-ATPase with higher expression in hypocotyls and leaves^78^; and
ix) *LHA4*, which encodes for a plasma membrane H^+^-ATPase with higher expression in roots and hypocotyls^78^.

As an example, we then focused on *LHA4* in tomato and by matching its sequence by BLAST^79^ against the NCBI nt database, we found that *LHA4* is homologous to *AHA2* in *A*. *thaliana*. *AHA2*’s overexpression has been suggested to improve salinity tolerance^80^. AHA2 was also shown to be phosphorylated upon salt stress^81^. However, and despite the growing amount of evidence, little is known about the role of AHA2 (Arabidopsis) in salinity stress. This example demonstrates how DES-TOMATO can facilitate an easy review of dictionary terms associated with a term of interest.

**Example 3**. Using *“Explore Hypotheses”* to *demonstrate how topic*-*specific hypothesis can be generated and tested*.

**Figure 4:**
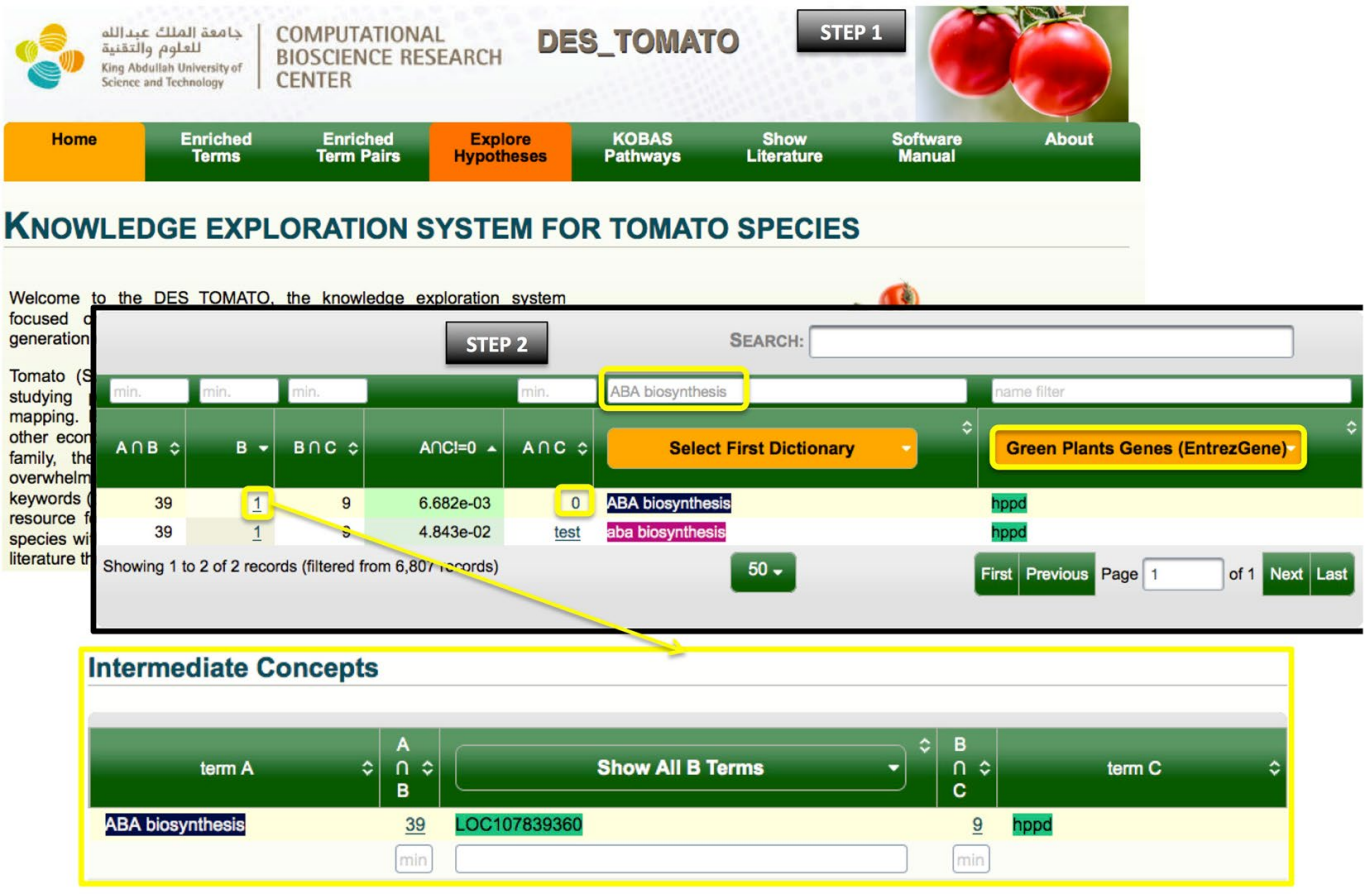
A simple demonstration of how a use “Explore hypotheses”. Boxed in yellow are the criteria used to direct or test the hypotheses generated.

Plant growth is affected by various abiotic stress conditions in which abscisic acid (ABA) biosynthesis is a major hub. To generate a hypothesis on this topic, we used the “Explore Hypotheses” tool, which opens a page with two columns listing associated enriched terms from all dictionaries (Fig. 4, Step 1). The first dictionary (term A) was filtered with ‘ABA biosynthesis’ while for the second dictionary (term C), we selected “Green Plants Genes” dictionary from the drop-down menu, after which we clicked ‘test’ for *hppd* (Fig. 4, Step 2). This generated a hypothesis that *hppd* may be linked to ABA biosynthesis via the linking term LOC107839360 (term B), also known as carotenoid 9,10(9’,10’)-cleavage dioxygenase 1-like.

The *hppd* gene encodes the enzyme p-hydroxyphenylpyruvate dioxygenase that acts as an oxireductase on pyruvate carriers. To our knowledge, current literature provides no direct link between p-hydroxyphenylpyruvate dioxygenase and ABA biosynthesis. But interestingly, pyruvate carriers have recently been implicated in ABA signaling^82^. In Arabidopsis, the putative mitochondrial pyruvate carrier, NRGA1, is a negative regulator of guard cell ABA signaling through the alleviation of ABA effect. This suggests that NRGA1 is responsible for the maintenance of optimal stomatal aperture during drought stress^82^. Here we show that by using “Explore Hypotheses”, we were able to conjecture that p-hydroxyphenylpyruvate dioxygenase (encoded by *hppd*) may act on the NRGA1 pyruvate carrier and consequently may indirectly interact with ABA. Further studies are required to validate this hypothesis.

**Example 4**. *Exploring S*. *lycorpersicum enriched pathways using “KOBAS Pathways”*.

**Figure 5:**
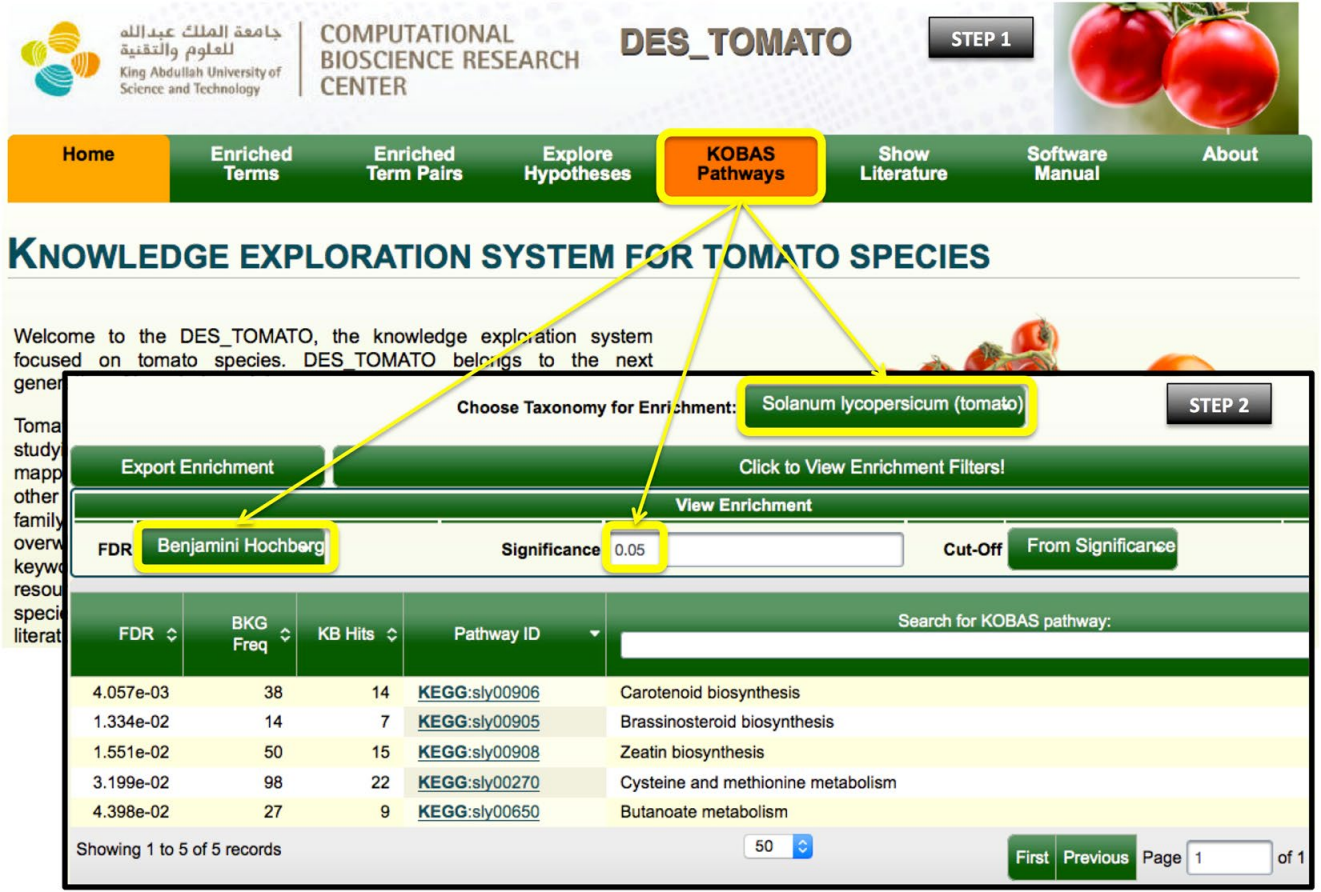
A simple demonstration of how *S*. *lycorpersicum* enriched pathways can be explored using “KOBAS Pathways”. Boxed in yellow are the criteria adapted for this exploration process.

Here we demonstrate how users can easily access the supplementary information from the KOBAS database^52^ using DES-TOMATO. First, we clicked on “KOBAS Pathways” (top menu) and selected ‘*Solanum lycorpersicum’* from the “taxonomy for enrichment” drop-down menu. By selecting Benjamini-Hochberg correction and a significance level of 0.05 (View Enrichment Filters button), we obtain five enriched pathways (Fig. 5): (1) carotenoid biosynthesis; (2) brassinosteroid biosynthesis; (3) zeatin biosynthesis; (4) cysteine and methionine metabolism; and (5) butanoate metabolism. All of these pathways have been described in tomato as major contributors to plant and fruit development, fruit ripening and pathogen-resistance^83–89^. To further understand why these pathways are statistically enriched in tomato literature, we provide a brief and simple description for each.

1. Carotenoid biosynthesis. Carotenoids are colored pigments present in all plant tissues, and their formation is highly regulated. Lycopene is the major carotenoid in tomato. During fruit ripening, lycopenes’ concentration increases enormously^90^. The regulation of carotenoids biosynthesis in tomato and other major genes (e.g. phytoene synthase - *Psy* and and phytoene desaturase -*Pds*) that are involved in this process have been extensively studied^84, 90–92^;
2. Brassinosteroid biosynthesis. Brassinosteroids are steroidal hormones that are essential for plant growth and development, and are also involved in stress-response mechanisms^93, 94^. Castasterone is a precursor in the brassinosteroid biosynthesis pathway, which is the product of a cytochrome P450-catalyzed conversion reaction from 6-deoxocastasterone. The cytochrome P450 and its *Dwarf* encoding gene have been extensively studied in tomato fruit development^85, 95–97^;
3. Zeatin biosynthesis. Zeatins are plant-growth hormones that belong to the cytokinins family. They regulate cell division and expansion and delay senescence. In tomato, changes in root-synthesized zeatins have been implicated in stress-responses^86, 88^, and fruit development^89^;
4. Cysteine and methionine metabolism. Methionine is an essential amino acid, and is the precursor of ethylene. Ethylene is a plant hormone that is involved in several processes in plant life-cycle including seed germination, root hair development, flower senescence and fruit ripening^87^. In tomato, biosynthesis of ethylene has been extensively studied due to its importance in controlling fruit ripening^83, 87, 98^;
5. Butanoate metabolism. Gamma-aminobutyric acid (GABA) is a non-protein amino acid, and a major plant-growth regulator^99^. GABA levels undergo drastic fluctuations during fruit development, by increasing during the mature green stage, and rapidly decreasing during the ripening stage^100, 101^.

## Discussion

### General Comments

Text-mining will not replace other types of computational data analysis in the biomedical field, the same way computational methods in general will not replace laboratory experiments and clinical research. However, text-mining should be considered as complementary to other (experimental and computational) approaches. The information obtained through text-mining, in many cases, cannot be obtained through other means in any simple manner^102^. Indeed, text-mining approaches have been deployed to complement other lines of investigation or as stand-alone tools for gaining quick insights. There are several reports where text-mined data alone were used to correctly infer links between concepts, e.g. Smalheiser and Swanson correctly inferred a link between Alzheimer’s disease and indomethacin^103, 104^ and Wren *et al*. correctly inferred a link between chlorpromazine and the progression of cardiac hypertrophy^105^. Text-mining was also used in conjunction with gene expression analysis to show that sphingosine 1-phosphate independently regulates glioblastoma cell invasiveness through urokinase-type plasminogen activators^106, 107^. Similarly, text-mining was also used with other types of data-mining to successfully identify disease genes in Wilms’ tumor^108^. Moreover, text-mining was successfully used to identify protein-protein interactions (see e.g. refs 36 and 37), transcription factor associations^38^, and methylated genes in various diseases and species^39, 109^. Thus, text-mining approaches are increasingly playing a role in a number of biomedical problems^110^ from pharmacogenomics^111^ (for the extraction of relations between drugs, genes and diseases), to precision medicine and drug repositioning^26^.

### Limitations

DES-TOMATO generally has the same limitations as other existing text-mining-based resources. Here we list some of the most common constraints: 1/text-mining-based resources are confined to information presented in electronically available documents; 2/some documents are protected by copyright from text-mining; 3/all text-mining systems are far from being able to extract all useful information from available texts; 4/peer-reviewed literature contains errors that are often propagated in different articles and automated text-mining information extraction cannot correct for such errors. This field undoubtedly requires significant improvements. Additionally, an association in DES-TOMATO does not specify the type of relationship among the extracted pairs of entities, e.g. co-occurrence of terms does not necessarily imply direct or physical interaction between paired terms.

Coverage is also affected by the common practice of authors to report only on what are deemed as the most relevant data. For example, papers reporting on genomic studies related to gene expression data, describe only a handful of genes in the text, while the bulk of experimental results are deposited separately from the published articles. In DES-TOMATO, dictionaries cover 3,050 Solanaceae species and all of their 300,973 non-redundant genes. This was necessary in order to maximize coverage of the tomato genes and their potential homologs. However, only 297 species (10%) and 2,994 genes (1%) were enriched in the text, which is not surprising.

The question now becomes, given the constraints imposed on the information that can be extracted from text, is it even worth using it? We believe the answer is yes, for the very fact that the type of information in the published scientific literature in the vast majority of cases conveys what researchers considered the most important facts regarding the topic of interest. The vast majority of scientific studies start by reviewing literature on the topic of interest and not by delving directly into the analysis of experimental data. However, due to limitations in terms of coverage and sometimes uncertainty of the quality of automatically extracted information through text-mining, the resulting data presented to the user are mainly advisory, aimed to guide exploration and draw attention to linked concepts. Domain knowledge and expertise are required for the interpretation of linked concepts, equally as they are required for the interpretation of experimental results.

## Concluding Remarks

Recent biotechnological advances have unleashed a tsunami of scientific literature that has become overwhelming for researchers. Even for the topic-specific literature insight, the volume of information is huge. To meet this challenge, we developed the DES-TOMATO KB that is focused on tomato species and its close relatives. DES-TOMATO performs the critical task of rapidly and comprehensively sifting through more than 20 thousands topic-specific publications and extracting relevant knowledge, both established and possibly novel. The current release comprises mined text elements from 22,647 tomato-related articles, in which 52,886 statistically enriched terms from 26 relevant dictionaries were identified, together with 1,388,952 statistically enriched pairs of these terms.

DES-TOMATO has various tools that enable users to perform complex tasks including querying for enriched terms or pairs of terms, building and testing hypotheses based on transitive associations, identifying enriched KOBAS pathways based on list of genes and proteins identified in the KB corpus. Using the network viewer, results can be visualized and further developed by successively expanding upon terms of interest using selected dictionaries; thus, offering a highly flexible exploration experience. In addition, publications that substantiate enrichment of a term or an association are readily accessible to the user. DES-TOMATO exceeds other discovery platforms in plant sciences (such as SGN and HRGRN), through the use of a literature text-mining methodology that enables: 1) computational assignment of terms-to-publication associations (i.e. independent of gene identifiers); 2) very comprehensive coverage of information not easily or not at all available in other tomato-related databases; 3) straightforward and regular updates with new publications to ensure the KB remains current and relevant.

DES-TOMATO is a unique information/knowledge exploration system in plant sciences. It was built to explore and generate useful information using a broad set of topic-related dictionaries that provide the user the flexibility to examine various questions. DES-TOMATO also provides a user-friendly interface, and an extensive instructional material to facilitate the navigation through the KB. Altogether, we hope that DES-TOMATO will be a useful tool for supporting tomato-related research questions^112^.

## Electronic supplementary material

Supplementary Material
