# Supplementary material for: DES-TOMATO: A Knowledge Exploration System Focused On Tomato Species
Source: Sci Rep. 2017 Jul 20;7:5968. doi: 10.1038/s41598-017-05448-0 (PMC5519719; doi:10.1038/s41598-017-05448-0)
Supplement: Supplementary file 1 — Supplementary Material [file 41598_2017_5448_MOESM1_ESM.pdf]

Supplementary Material

DES-TOMATO: A Knowledge Exploration System Focused On Tomato Species

Adil Salhi[1#], Sónia Negrão[2#], Magbubah Essack[1#], Mitchell J.L. Morton[2], Salim Bougouffa[1], Rozaimi Razali[1], Aleksandar Radovanovic[1], Benoit Marchand[3], Maxat Kulmanov[1], Robert Hoehndorf [1,4], Mark Tester[2], Vladimir B. Bajic[1,4]*

[1]King Abdullah University of Science and Technology (KAUST), Computational Bioscience Research Center (CBRC), Thuwal 23955-6900, Kingdom of Saudi Arabia, [2]King Abdullah University of Science and Technology (KAUST), Division of Biological and Environmental Sciences and Engineering, Thuwal, 23955-6900, Saudi Arabia, [3]New York University, Abu Dhabi, UAE,[4]King Abdullah University of Science and Technology (KAUST), Computer, Electrical and Mathematical Sciences and Engineering Division (CEMSE), Thuwal 23955-6900, Kingdom of Saudi Arabia

*To whom correspondence should be addressed, [#]shared first authors

To show usefulness of the enrichment measure we use in DES-TOMATO, we additionally provide the distribution of high similarity pairs across FDR rank. Figure S1 a, b and c demonstrates that the higher the FDR rank of a gene pair, the more likely it would have a high rank based on semantic similarity.

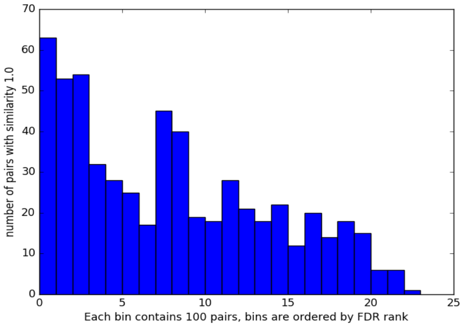

a

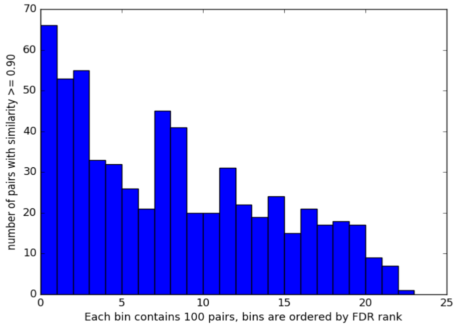

b

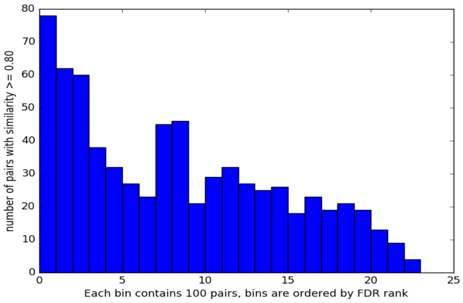

c

Figure S1 a,b,c show the distribution of gene pairs having semantic similarity (1.0, >=0.9, and >=0.8) respectively, according to our measure of ranking gene pairs extracted from text, namely FDR.

Table S1. Examples of gene-gene associations identified in KB with different semantic similarity scores.

Gene Symbol/Description/Common Annotations	Gene Symbol/Description/Common Annotations	Semantic Similarity Score
TAPG1<br>**ID: 544004 polygalacturonase 1 [*Solanum lycopersicum* (tomato)]**<br><br>"polygalacturonase activity";"molecular_function";"GO:0004650"<br>"carbohydrate metabolic process";"biological_process";"GO:0005975"	Cel2<br>**ID: 543996 endo-1,4-beta-glucanase precursor [*Solanum lycopersicum* (tomato)]**<br><br>"hydrolase activity, hydrolyzing O-glycosyl compounds";"molecular_function";"GO:0004553"<br>"carbohydrate metabolic process";"biological_process";"GO:0005975"	**Sim = 0.89**
cemA<br>**ID: 3950384 chloroplast envelope membrane protein [*Solanum lycopersicum* (tomato)]**<br><br>"structural constituent of ribosome";"molecular_function";"GO:0003735"<br>"ribosome";"cellular_component";"GO:0005840"<br>"translation";"biological_process";"GO:0006412"	psbB<br>**ID: 3950388 photosystem II CP47 chlorophyll apoprotein [*Solanum lycopersicum* (tomato)]**<br><br>"structural constituent of ribosome";"molecular_function";"GO:0003735"<br>"intracellular";"cellular_component";"GO:0005622"<br>"ribosome";"cellular_component";"GO:0005840"<br>"translation";"biological_process";"GO:0006412"<br>"rRNA binding";"molecular_function";"GO:0019843"	**Sim = 0.86**
Cel8<br>**ID: 543583 endo-beta-1,4-D-glucanase [*Solanum lycopersicum* (tomato)]**<br><br>"hydrolase activity, hydrolyzing O-glycosyl compounds";"molecular_function";"GO:0004553"<br>"extracellular region";"cellular_component";"GO:0005576"<br>"carbohydrate metabolic process";"biological_process";"GO:0005975"<br>"carbohydrate binding";"molecular_function";"GO:0030246"	cel7<br>**ID: 544125 endo-1,4-beta-D-glucanase [*Solanum lycopersicum* (tomato)]**<br><br>"hydrolase activity, hydrolyzing O-glycosyl compounds";"molecular_function";"GO:0004553"<br>"carbohydrate metabolic process";"biological_process";"GO:0005975"	**Sim = 0.77**
psbA<br>**ID: 3950408 photosystem II protein D1 [*Solanum lycopersicum* (tomato)]**<br><br>"photosystem I";"cellular_component";"GO:0009522"<br>"thylakoid";"cellular_component";"GO:0009579"<br>"photosynthetic electron transport in photosystem II";"biological_process";"GO:0009772"<br>"integral component of membrane";"cellular_component";"GO:0016021"<br>"photosynthesis, light reaction";"biological_process";"GO:0019684"<br>"plasma membrane light-harvesting complex";"cellular_component";"GO:0030077"<br>"electron transporter, transferring electrons within the cyclic electron transport pathway of photosynthesis activity";"molecular_function";"GO:0045156"	ndhF<br>**ID: 3950404 NADH-plastoquinone oxidoreductase subunit 5 [*Solanum lycopersicum* (tomato)]**<br><br>"photosystem I";"cellular_component";"GO:0009522"<br>"thylakoid";"cellular_component";"GO:0009579"<br>"photosynthesis";"biological_process";"GO:0015979"<br>"integral component of membrane";"cellular_component";"GO:0016021"	**Sim = 0.68**
